# Feasibility, safety, and adherence of home-based exercise interventions in people diagnosed with cancer: a systematic review

**DOI:** 10.1007/s11764-025-01778-5

**Published:** 2025-03-22

**Authors:** Jing Jing Su, Petr Winnige, Katerina Chamradova, Filip Dosbaba, Katerina Batalikova, Rose Lin, Varsamo Antoniou, Garyfallia Pepera, Ladislav Batalik

**Affiliations:** 1 School of Nursing, Tung Wah College, Hong Kong, China; 2 Department of Rehabilitation, University Hospital Brno, Brno, Czech Republic; 3 Department of Physiotherapy and Rehabilitation, Faculty of Medicine, Masaryk University, Brno, Czech Republic; 4 Department of Rehabilitation and Sports Medicine, Second Faculty of Medicine, Motol University Hospital, Charles University, Prague, Czech Republic; 5 Department of Rehabilitation, Faculty of Medicine, Masaryk University, Brno, Czech Republic; 6 Elaine Hubbard Center for Nursing Research On Aging, School of Nursing, University of Rochester Medical Center, Rochester, NY USA; 7 Clinical Exercise Physiology and Rehabilitation Research Laboratory, Physiotherapy Department, School of Health Sciences, University of Thessaly, Lamia, Greece; 8 Department of Public Health, Faculty of Medicine, Masaryk University, Brno, Czech Republic

**Keywords:** Home-based exercise, Cancer survivors, Feasibility, Safety, Adherence, Physical activity, Adverse events

## Abstract

**Purpose:**

This systematic review aims to evaluate the feasibility, safety, and adherence of home-based exercise interventions in people diagnosed with cancer. The primary research question is: *Are home-based exercise interventions safe and feasible for people diagnosed with cancer?*

**Methods:**

A comprehensive search of databases including PubMed, EMBASE, and Cochrane Library was conducted in January 2025, focusing on randomized controlled trials (RCTs) that involved home-based exercise interventions people diagnosed with cancer. Studies were included if they reported on safety, feasibility, and health-related outcomes. The Physiotherapy Evidence Database (PEDro) scale was used to assess study quality and risk of bias. Adverse events were categorized by severity, and feasibility which was determined based on recruitment, withdrawal, and adherence rates.

**Results:**

From 127 eligible studies involving 10,562 participants, the review found that home-based exercise interventions are generally safe, with less than 3.2% of participants experiencing exercise-related adverse events, most of which were minor. Feasibility was supported by an average recruitment rate of 50.1%, which was calculated as the proportion of eligible participants who consented to participate across the included studies. Additionally, the review found a withdrawal rate of 13.7%, and an adherence rate of 76.2%. However, significant variability in these rates were observed across the studies, highlighting the challenges in maintaining participant engagement.

**Conclusions:**

Home-based exercise interventions are feasible and safe for people diagnosed with cancer, with minor adverse events being the most common. However, there is a need for standardized protocols in reporting adverse events and better strategies to improve recruitment and adherence.

**Implications for cancer survivors:**

These findings support the integration of home-based exercise into standard cancer care, offering a practical and safe option for enhancing the health and well-being of cancer survivors. However, the successful implementation of these programs may require additional support from exercise professionals within primary care or community settings to ensure appropriate guidance and adherence. Personalized exercise programs, developed by qualified exercise professionals such as physiotherapists or clinical exercise physiologists, and improved reporting standards are essential to optimizing these interventions.

**Supplementary Information:**

The online version contains supplementary material available at https://doi.org/10.1007/s11764-025-01778-5.

## Introduction

Exercise training, particularly in home-based and unsupervised settings, is increasingly recognized as a valuable adjunct therapy in oncology, offering significant potential benefits for individuals living with cancer [1–4]. Over the past few decades, numerous trials have explored the role of home-based exercise in cancer care, demonstrating its capacity to positively impact a range of outcomes, including physiological, biological, functional, and patient-reported metrics [5–7]. Systematic reviews and meta-analyses have consistently highlighted the benefits of exercise during and after cancer treatment, showing improvements in fatigue, aerobic fitness, muscular strength, anxiety, body image, self-esteem, cognitive health, psychosocial distress, and overall quality of life [8, 9].

Despite the growing evidence for integrating exercise into cancer care, concerns persist regarding the safety of home-based physical exercise interventions, particularly when implemented without direct supervision in patients undergoing systemic cancer treatments. Recent instances, such as the premature discontinuation of exercise trials in chemotherapy-treated breast and lung cancer patients due to adverse events, underscore the need for a comprehensive understanding of the risks associated with unsupervised home-based exercise [10–12]. Additionally, the safety of these interventions remains uncertain in specific cancer populations, such as those experiencing cachexia and frailty [13].

Accurate assessment of exercise-related harms is crucial for guiding evidence-based decision-making in clinical practice, especially for home-based exercise programs. However, previous systematic reviews examining adverse events in this context have often faced limitations, including insufficient scrutiny of the quality of available evidence, potentially leading to biased conclusions [14].

Recognizing these critical gaps, we conducted a comprehensive systematic review of clinical trials focusing on home-based exercise or physical activity interventions in people diagnosed with cancer. Our primary objectives were to evaluate the number, type, and severity of adverse events to establish a safety profile, while also addressing gaps in current evidence related to populations that may be at higher risk of adverse effects when exercising without supervision. We also aimed to assess recruitment, withdrawal, and adherence rates to determine the feasibility of such interventions as well as evaluate the quality of adverse event reporting and examine the circumstances leading to trial withdrawal, discontinuation, or patient withdrawal in both intervention and control arms.

By addressing these objectives, our review sought to provide a comprehensive understanding of the safety and feasibility of home-based exercise training in cancer care. Through a careful examination of the available evidence, we aimed to inform and optimize the integration of manageable, home-based exercise interventions into standard care practices for people diagnosed with cancer.

## Methods

This study follows the guidelines outlined in the Preferred Reporting Items for Systematic Reviews and Meta-Analyses statement, including its extensions for reporting harms [15]. The review protocol was registered on PROSPERO (CRD42023482624) on November 24, 2023.

### Search strategy and selection criteria

Eligibility criteria were determined based on the Participants, Intervention, Comparator, and Outcome (PICO) framework [16].

### Participants

Randomized controlled trials (RCTs) and single-arm trials involving male and female participants (aged ≥ 18 years) with cancer, either preparing for, undergoing or having completed treatment were included.

### Intervention

In this study, RCTs were defined as comparative investigations aimed at evaluating the safety and effectiveness of home-based exercise or physical activity interventions, while single-arm trials were defined as studies focusing solely on evaluating feasibility and acceptability metrics. Exercise was characterized as any planned, structured, and repetitive physical activity intended to enhance or maintain fitness, performance, or health. Exercise modes were classified into categories: aerobic, resistance, physical activity, or other [exercises not specifically categorized as aerobic or resistance (e.g., mind–body practices, which focus on mental focus, controlled breathing, and body movements to enhance mind–body awareness) or common lymphedema treatments (e.g., flexibility, passive exercises, or exercises performed without resistance)]. Participants were randomly assigned to either the intervention group or a usual care group. The interventions had to be designed to increase exercise or physical activity levels and improve health-related outcomes, such as quality of life. Trials were included regardless of the mode of delivery, duration, or intensity of the intervention. Studies combining exercise with other interventions, such as dietary or lifestyle changes, were excluded if the effects of exercise outcomes could not be isolated. Interventions conducted before, during, or after cancer treatment were all considered eligible for inclusion.

### Comparators

For inclusion, studies had to incorporate a usual care or control group. Studies featuring multiple intervention arms were considered eligible if at least one group was assigned to usual care or control.

### Outcomes

The outcomes of interest in this study included safety, feasibility, and various health-related measures. Safety was assessed by identifying adverse events, defined as any negative or unintended medical or health-related occurrences during the study. These events were classified as either non-exercise-related or exercise-related, with severity categorized using the Common Terminology Criteria for Adverse Events, Version 4, ranging from grade 1 (minor symptoms or asymptomatic) to grade 5 (death) [17]. Serious adverse events were those that required hospitalization, led to significant disability, were life-threatening, or resulted in death. Health-related withdrawals, such as those due to illness or cancer recurrence, were also considered adverse events. Withdrawals for non-health-related reasons (e.g., time constraints, travel issues) were excluded. Cases where the severity of an adverse event was not reported but led to study withdrawal, or a participant withdrew for unspecified health reasons, were classified as grade 3. Feasibility and acceptability were assessed by examining recruitment rates, withdrawal rates, and exercise adherence rates. Recruitment rates were calculated as the proportion of eligible participants who consented to participate, while withdrawal rates reflected the percentage of participants who did not complete the study. Exercise adherence was measured by the percentage of scheduled exercise sessions that participants completed.

### Search methods

Search methods included an electronic literature search carried out in January 2025 using databases such as PubMed, EMBASE, Scopus, Cochrane Library, CINAHL, and EBSCOhost. The search was designed to identify studies on the impact of home-based exercise interventions published in English from inception. The search strategy employed keywords related to home-based exercise, physical activity, and cancer, as outlined in Supplementary Table 1. Relevant articles were initially selected based on these keywords following the literature search. Two independent reviewers (LB & PW) manually evaluated the articles based on their titles and abstracts. In the next step, the full texts of the selected articles were reviewed for relevance according to the inclusion criteria. Any disagreements between the reviewers were resolved through discussion or consultation with a third reviewer (KCH). When a study had multiple publications, the most comprehensive version with systematic data was chosen for analysis. Additionally, the authors of the studies were ted when further information was needed.

### Study quality and risk of bias

The methodological quality of each RCT was independently evaluated by two investigators (LB and PW) using the Physiotherapy Evidence Database (PEDro) scale, a validated and reliable tool for assessing risk of bias and overall quality in RCTs [18]. Additionally, the Cochrane Risk of Bias 2 (ROB 2) tool was used to identify and assess potential biases in all included RCTs. ROB 2 evaluates domains such as bias arising from the randomization process, deviations from intended interventions, missing outcome data, measurement of the outcome, and selection of the reported result. A summary of PEDro scores is presented in the main text, while detailed ROB 2 assessments, including judgments for each domain, are included in Supplementary Table 7 and Supplementary Table 8. The PEDro scale includes 11 items—covering eligibility criteria, random allocation, allocation concealment, baseline comparability between groups, blinding of subjects, therapists, and assessors, attrition, intention-to-treat analysis, between-group statistical comparisons, and reporting of outcomes. The total PEDro score ranges from 0 to 10 points, with the first item (eligibility criteria) not contributing to the total score. RCTs scoring 6 or higher were deemed high quality, while those scoring below 6 were classified as low quality. Any discrepancies in ratings were resolved through discussion, with a third reviewer (FD) consulted if needed.

The quality of adverse event reporting was evaluated based on adherence to the CONSORT statement extension for reporting harms (Supplementary Table 2). This assessment used a 16-item scoring system adapted from prior studies [19, 20]. Each item received a score of ‘1’ if it was clearly reported and a score of ‘0’ if it was either unclear or not reported. The overall quality score was determined by summing the scores of all items, with each item weighted equally. Two authors (LB & PW) independently conducted these assessments.

### Data extraction

The titles and abstracts of all articles identified through electronic database searches were independently reviewed by two authors (LB & PW). In cases of disagreement, a third author (KCH) was consulted to reach a consensus. Full-text versions of relevant records were then retrieved and further screened against the eligibility criteria. Data on study characteristics, participant demographics, intervention details, and assessed outcomes from the included articles were systematically extracted into a tabular format using predefined data fields.

Feasibility was evaluated by calculating the study's recruitment rate, withdrawal rate, and exercise adherence rate, all expressed as percentages. To ensure a comprehensive analysis and account for potential data skewness, the median, interquartile range, and minimum and maximum rates were reported. The feasibility of the exercise intervention was defined based on the following a priori clinically relevant cut-offs: a recruitment rate of over 25%, a withdrawal rate of less than 25%, and an adherence rate where more than 75% of participants reported high adherence [21]. These thresholds were established based on previous literature to determine the practicality and success of the intervention [22, 23].

## Results

**Fig. 1:**
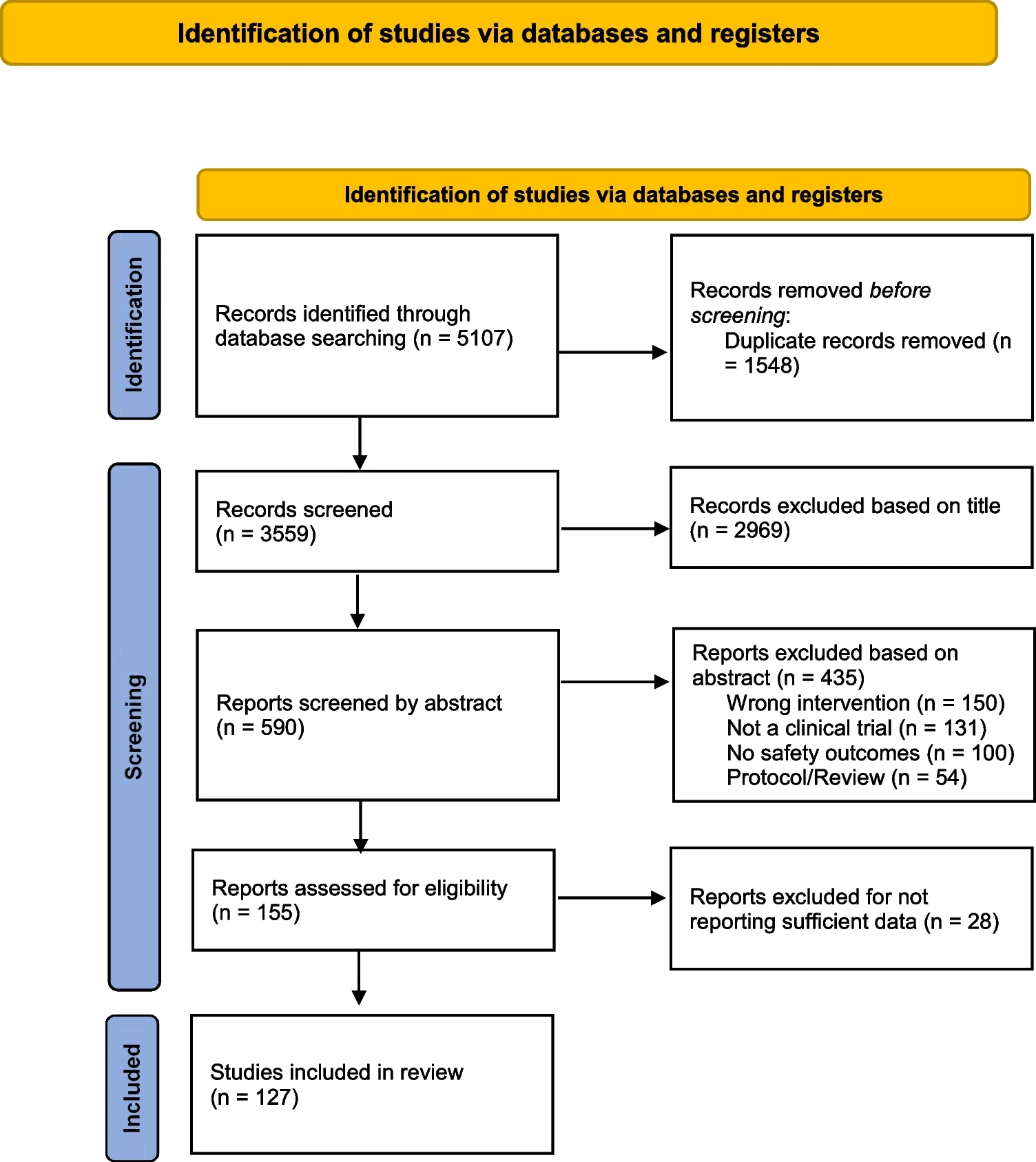
PRISMA flowchart of the selection process

A database and meta-search engine search were performed and identified 5107 records. After screening the titles and abstracts, it was found that 2969 publications did not meet the inclusion criteria. Of the 590 full-text publications, 463 records were excluded. Finally, 127 publications met the inclusion criteria for this systematic review [10–12, 24–147]. Figure 1 provides an overview of the study flow process.

### Description of eligible trials

Characteristics of the eligible trials are summarized in Table 1 and presented in full detail in Supplementary File 3. The eligible trials included a total of 12,61 allocated participants, with a median (IQR) sample size of 60 (60.5). Most (n = 120; 94.5%) trials were described as RCTs, seven (5.5%) were single arm trials [24, 32, 60, 71, 103, 104, 134]. The most commonly studied tumor sites were breast (n = 47; 397%) [10, 12, 31–33, 37, 39, 41, 46, 49, 51, 57, 59, 62, 70, 71, 76, 77, 80, 85–88, 92, 93, 96–98, 100, 101, 104–108, 111, 115, 116, 119, 121, 125, 126, 131, 133, 134, 136, 137] and mixed sites (n = 22; 17.3%) [28, 30, 42, 43, 45, 63, 66, 78, 79, 90, 94, 95, 109, 113, 117, 120, 122, 124, 127, 129, 143]. Trial participants had a mean (SD) age of 58 (9) years. While 5 (3.9%) trials had safety as the primary outcome [30, 72, 73, 130, 139], 23 (18.1%) trials focused on feasibility [12, 31, 35, 41, 53, 54, 63, 70, 74, 75, 86, 89, 107, 109, 111, 119, 120, 124, 126, 130, 137, 140, 145]. The majority of studies included in this review originated from North America, accounting for 48 studies (37.8%), followed by Europe with 35 studies (27.6%). Australia contributed 23 studies (18.1%), and 18 studies (14.2%) came from Asia. A smaller proportion of studies, 3 in total (2.4%), originated from South America, indicating a more limited research focus in this region.

**Table 1:** Characteristics of the eligible trials (n = 127)

Trial design	
Sample size, median (IQR)	60 (60.5)
1Non-small trials, n (%)	16 (12.6)
Allocation method	
Randomization, n (%)	119 (93.7)
Single arm, n (%)	8 (6.3)
Participants	
Age, mean (SD)	58.7 (8.7)
Cancer, n (%)	
Breast Cancer	47 (37.0)
Mixed	22 (17.3)
Prostate Cancer	18 (14.2)
Haematopoietic or lymphoid tissues	10 (7.8)
Lung	11 (8.5)
Colorectal Cancer	7 (5.5)
Gastrointestinal cancer	6 (4.7)
Endometrial cancer	3 (2.4)
Metastatic solid cancer	2 (1.5)
Gliomas	1 (0.8)
Therapy stage, n (%)	
Ongoing systemic therapy	59 (46.5)
Completed therapy	68 (53.5)
Ongoing systemic therapy, n (%)	
Chemotherapy	19 (32.2)
Combination	18 (30.5)
Androgen deprivation therapy	12 (20.3)
Allogenic stem cell transplantation	4 (6.8)
Surgery	6 (10.2)
Exercise intervention characteristics	
Modality, n (%)	
Aerobic	20 (15.8)
Physical Activity	21 (16.5)
Resistance	10 (7.9)
Mind–Body	7 (5.5)
Combined	69 (54.3)
1 Defined as > 100 participants per trial arm	

The majority of interventions provided various forms of guidance, feedback, and counseling aimed at resolving queries, monitoring and tailoring exercise and physical activity progression, and addressing emotional and health status. The most common method of guidance was delivered via telephone, used in 35% of the studies [27, 31, 33, 35, 38, 39, 41, 42, 47, 49, 50, 53–56, 59, 64, 65, 67, 70, 75, 80–82, 88, 89, 94–96, 101, 102, 104, 106, 116, 120, 125, 128, 130, 136, 138, 141, 143, 144, 147]. In-person and group-based interventions were used in 20% of the studies [24–27, 33, 38, 41, 48, 51, 55, 69, 80, 85, 87, 90, 93, 98, 103, 106, 110, 114–116, 122, 137]. Text message-based feedback was implemented in 9% of the studies [29, 34, 53, 74, 75, 82, 94, 96, 101, 105, 108, 146]. One study utilized a video-based model [81]. Several studies employed a combination of multiple intervention types [27, 33, 38, 41, 53, 55, 75, 80–82, 96, 101]. Additionally, 47% of the studies were self-directed or did not report the specific guidance or counseling methodology used.

### Description of the exercise interventions

The median (IQR) exercise intervention length was 12 (14) weeks, and 58 (46%) trials evaluated aerobic, physical activity and resistance exercise modality [10–12, 28, 29, 31, 36, 40, 44, 45, 48, 50, 53, 59, 64, 70–72, 74, 80, 81, 85, 87, 88, 92, 95, 100–102, 104, 105, 108, 110, 115, 123, 124, 126–128, 133, 135–141, 143, 145, 147]. Just over a half (n = 69; 54%) of the trials evaluated exercise in combination with other interventions; The exercise interventions varied across trials, with some notable patterns. For example, 47 (37%) trials involved an aerobic + resistance combination [24–27, 33–35, 39, 41, 42, 46, 47, 49, 51, 55, 57, 58, 62, 63, 65, 66, 69, 75–77, 82–84, 90, 91, 94, 96, 97, 99, 106, 112–114, 117–120, 122, 130–132, 142], while 8 (7%) trials included a physical activity + resistance combination [52, 54, 78, 93, 98, 109, 116, 125]. Additionally, 4 (3%) trials incorporated aerobic + resistance + flexibility exercises [37, 38, 68, 103] and one trial included resistance + balance training [43]. A smaller number of trials, involved more specialized interventions like physical activity + inspiratory muscle training [56, 144] and aerobic + balance, respectively [60]. Furthermore, one trial reported incorporating aerobic + inspiratory muscle training [73], and a more comprehensive approach with aerobic + resistance + flexibility + balance [34]. Of the trials, 9 (7%) reported including combination mind–body exercises [30, 32, 61, 67, 79, 86, 107, 121, 146].

Exercise contraindications were reported in 21 trials (17.5%), totaling 20 different conditions. The most common contraindications included pain (n = 12), fever (n = 9), thrombocytopenia (n = 7), anemia (n = 7), and infection (n = 6). Other reported conditions were dyspnea (n = 5), dizziness (n = 4), nausea (n = 4), orthostatic hypotension (n = 3), hypertension (n = 3), weakness (n = 3), anxiety (n = 2), tachycardia (n = 2), palpitations (n = 2), acute bleeding (n = 1), arrhythmias (n = 1), abnormal cardiac response (n = 1), vomiting (n = 1), vertigo (n = 1), and headache (n = 1).

Digital wearable devices were utilized to share and monitor exercise and physical activity, track exercise metrics, and provide feedback, enhancing the effectiveness and adherence of interventions. A total of 52 studies (41%) utilized digital wearable devices during the exercise. The most frequently used device was the pedometer, implemented in 24 (19%) studies [28, 31, 35, 42, 48, 52, 60, 65, 69, 73, 79, 81, 85, 88, 90, 93, 94, 96, 102, 105, 112, 123, 125, 126]. Heart rate monitors were used in 16 (13%) studies [26, 27, 36, 39, 53, 58, 75, 96, 101, 114, 118, 136, 140, 145, 147]. Fitbit tracking device, was used in 8 (7%) studies [24, 27, 34, 56, 63, 74, 101, 104]. Accelerometers were implemented in 4 (3%) studies [29, 44, 87, 89] and in one study the Xbox 360 Kinect system was applied [130].

### Risk of bias and overall quality

The risk of bias and overall quality of the RCTs included in the systematic review are summarized based on the PEDro scale (Supplementary Table 4). Out of the 127 trials assessed, 81 (64%) were rated as high quality, with a PEDro score of 6 or higher [10, 24–27, 29, 30, 32, 33, 36, 37, 40, 43, 46, 47, 49–53, 55, 56, 58, 60, 62, 64–67, 70, 72–74, 76–78, 80–83, 85, 87, 89–92, 94–97, 99–105, 107, 108, 110, 112–116, 126–132, 134, 137–139, 141–143, 145, 146]. Conversely, 46 (36%) trials were classified as low quality, scoring below 6 [11, 12, 12, 28, 31, 34, 35, 38, 39, 41, 42, 44, 45, 48, 54, 57, 59, 61, 63, 68, 69, 71, 75, 79, 84, 86, 88, 93, 98, 106, 109, 111, 117–125, 133, 135, 136, 140, 144, 145]. No trials were free of potential bias, but the most common factors contributing to lower quality ratings were the lack of blinding of participants and therapists and the failure to achieve adequate allocation concealment. These findings underscore the ongoing challenges in maintaining rigorous methodological standards in clinical research.

### Recruitment, withdrawal, and exercise adherence

Recruitment, withdrawal, and adherence rates are summarized in Table 2. The median recruitment rate across studies was 49% (range 8–100%), with 115/127 studies (90.6%) reporting recruitment rates. Of these, 17/115 (13.4%) did not meet the recruitment rate threshold (> 25%), with rates ranging from 7.9% to 24.6% [12, 30–32, 34, 36, 56, 89, 93, 95, 96, 108, 115, 120, 122, 128, 135, 146]. Most of these studies focused on breast cancer (6/17, 35.7%) and mixed cancer sites (4/17, 23.5%), with 64.7% (11/17) including participants who had completed cancer treatment. Recruitment rates varied based on exercise mode, with aerobic exercise studies showing the lowest rates (median 46%) and mind–body exercise studies showing the highest rates (median 61%). Recruitment was higher in studies with self-management approaches (median 60%) compared to specialist-led interventions (median 41%).

**Table 2:** Recruitment, withdrawal, and exercise adherence by exercise mode, treatment status, supervision, and intervention duration

	Recruitment rate (%) Median (minimum, maximum [IQR])	Withdrawal rate (%) Median (minimum, maximum [IQR])		Adherence rate (%) Median (minimum, maximum [IQR])
		Intervention	Usual care	
Overall	49 (8, 100 [41]), n = 115	13 (0, 38 [13]), n = 121	10 (0, 44 [13]), n = 113	79 (28, 100 [19]), n = 94
Exercise mode				
Aerobic exercise	46 (13, 100 [32]), n = 18	11 (0, 27 [11]), n = 19	8 (0, 35 [12]), n = 18	80 (60, 98 [13]), n = 18
Resistance exercise	36 (8, 79 [36]), n = 10	9 (0, 29 [9]), n = 10	7 (0, 30 [16]), n = 9	76 (55, 100 [12]), n = 6
Combined exercise	51 (11, 96 [42]), n = 61	14 (0, 38 [12]), n = 66	10 (0, 44 [11]), n = 62	78 (28, 100 [19]), n = 50
Physical activity	53 (21, 93 [36]), n = 19	9 (0, 29 [12]), n = 19	8 (0, 26 [12]), n = 18	79 (34, 100 [30]), n = 15
Mind–body exercise	61 (14, 95 [37]), n = 7	20 (4, 21 [2]), n = 7	9 (0, 25 [10]), n = 6	73 (49, 96 [20]), n = 5
Treatment status				
During treatment	48 (11, 100 [43]), n = 55	12 (0, 38 [14]), n = 58	10 (0, 35 [11]), n = 55	77 (31, 100 [19]), n = 49
Completed treatment	49 (8, 97 [38]), n = 60	14 (0, 32 [12]), n = 63	9 (0, 44 [15]), n = 58	79 (28, 100 [18]), n = 45
Guidance-feedback				
Specialist-led	41 (8, 97 [40]), n = 56	13 (0, 38 [12]), n = 62	10 (0, 30 [11]), n = 58	79 (28, 100 [17]), n = 46
Self-management	60 (14, 100 [35]), n = 59	14 (0, 29 [12]), n = 59	10 (0, 44 [15]), n = 55	77 (34, 100 [18]), n = 48
Intervention duration				
≤ 12 weeks	52 (11, 100 [38]), n = 73	13 (0, 31 [15]), n = 78	9 (0, 44 [13]), n = 72	79 (28, 100 [23]), n = 57
> 12 weeks	38 (8, 90 [36]), n = 42	14 (0, 38 [9]), n = 43	11 (0, 38 [11]), n = 41	78 (31, 100 [17]), n = 37
Sample age (mean)				
≥ 65 years	51 (8, 98 [42]), n = 88	14 (0, 38 [13]), n = 95	9 (0, 44 [12]), n = 88	78 (28, 100 [17]), n = 77
< 65 years	35 (23, 100 [35]), n = 27	12 (0, 27 [10]), n = 26	12 (0, 28 [12]), n = 25	81 (31, 100 [23]), n = 17
Study quality rating				
Low	52 (11, 100 [37]), n = 40	13 (0, 32 [15]), n = 42	13 (0, 44 [13]), n = 41	80 (34, 100 [18]), n = 34
High	47 (8, 97 [40]), n = 75	13 (0, 38 [11]), n = 79	9 (0, 30 [12]), n = 72	77 (28, 100 [19]), n = 60

n = values represent number of studies; Low quality: PEDro scale score of less than 6; high quality: PEDro scale score of 6 or higher

IQR: Interquartile range

The overall withdrawal rate across intervention groups was 13% (range 0–38%), with 121/127 studies (95.3%) reporting withdrawal rates. Among these, 12/121 (9.9%) exceeded the withdrawal rate threshold (< 25%), with rates ranging from 26.3% to 37.5% [24, 55, 59, 70, 85, 89, 96, 103, 106, 110, 135, 140]. Breast cancer was the most common cancer type in these studies (5/12, 41.7%). Studies evaluating resistance exercise had the lowest median withdrawal rate (9%), while mind–body exercise studies had the highest (20%). Withdrawal rates were slightly higher in studies with completed treatment (median 14%) compared to those conducted during treatment (median 12%). Health-related reasons were the most commonly cited cause for withdrawal, irrespective of intervention type or supervision approach.

Adherence rates were reported in 94/127 studies (74.0%), with a median adherence rate of 79% (range 28–100%). Of these, 34/94 (36.2%) did not meet the adherence rate threshold (> 75%), with adherence ranging from 28.0% to 74.0%. Breast cancer was the most commonly studied cancer type in these cases (11/34, 32.4%), followed by mixed sites (7/34, 20.6%) and prostate cancer (5/34, 14.7%). Adherence rates were highest in aerobic exercise studies (median 80%) and lowest in mind–body exercise studies (median 73%). Nearly half (16/34, 47.1%) of the studies that did not meet adherence thresholds provided various forms of guidance, feedback, and counseling [25–27, 41, 47, 55, 59, 64, 95, 103–105, 116, 120, 141, 143].

Notably, 75/127 studies (59.1%) satisfied all three feasibility criteria (recruitment, withdrawal, and adherence thresholds). Conversely, 52/127 studies (41.9%) did not meet at least one feasibility threshold [12, 24–28, 30, 32, 34, 36, 40, 41, 43, 44, 47, 52, 55–57, 59, 62, 64, 70, 79, 85, 89, 91–93, 95, 96, 99, 103–106, 108, 110, 115, 120–122, 124, 128, 131, 132, 135, 140, 141, 143, 145, 146], with adherence being the most common shortfall (34/52, 65.4%). Additionally, 11/127 studies (8.7%) did not meet two feasibility criteria [32, 55, 59, 89, 95, 96, 103, 115, 120, 135, 140].

### Adverse events in exercise participants

From trials included in this review, 80 (63%) explicitly reported that no adverse events had occurred [10, 24, 25, 27, 28, 31–33, 37, 39, 41, 43, 47, 51, 53–55, 59–61, 64, 66, 67, 69, 70, 72, 75–80, 82, 84–86, 88, 89, 91, 92, 97–101, 103, 104, 106, 107, 109–119, 123, 125–129, 132, 135–142, 144–147]. From the data of 2195 participants engaged in home-based exercise training, there were a total of 257 exercise-related adverse events out of 633 reported adverse events in 48 (38%) trials (Table 3) [11, 12, 30, 34–36, 38, 40, 42, 44–46, 48–50, 52, 56–58, 62, 63, 65, 68, 71, 73, 74, 81, 83, 87, 90, 93–96, 102, 105, 108, 120–122, 124, 130, 131, 133, 134, 143]. Grade 1 adverse events were the most common, totaling 210 events, and included musculoskeletal pain/myalgia/tendinitis (n = 99), musculoskeletal discomfort (n = 62), and increased fatigue (n = 24). Grade 2 adverse events were less frequent, with 28 events reported, including unspecified minor symptoms (n = 19) and gastrointestinal issues (n = 4). Grade 3 adverse events accounted for 19 cases, with the most significant being dizziness (n = 5) and deep vein thrombosis (n = 5). No grade 4 adverse events were reported. However, there were 37 grade 5 adverse events, all of which were deaths, though none were directly attributed to the exercise interventions.

**Table 3:** Adverse events categorized by the severity level are outlined for patients in both the exercise and usual care groups

AE Grade	Home-based exercise training (n = 2195)	Usual care (n = 2125)
	Exercise related adverse events / Total adverse events 257/632	Exercise related adverse events / Total adverse events 86/426
I	**Grade 1 adverse events 209/363**	**Grade 1 adverse events 79/226**
	Musculoskeletal pain/myalgia/tendinitis 99/164	Musculoskeletal pain/myalgia 33/89
	Musculoskeletal discomfort 62/100	Musculoskeletal discomfort 22/66
	Increased fatigue 24/41	Minor cardiac symptoms 1/31
	Musculoskeletal soreness 11/11	Increased fatigue 14/26
	Dyspnea 6/7	Musculoskeletal soreness 6/6
	Minor cardiac symptoms 5/35	Dyspnea 2/4
	Tendinitis 0/2	Tendinitis 1/3
	skin irritation ( accelerometer wear) 1/1	bronchitis 0/1
	Bruising 1/2	
II	**Grade 2 adverse events 28/70**	**Grade 2 adverse events 6/27**
	unspecified minor symptoms 19/37	unspecified minor symptoms 2/15
	gastrointestinal 4/14	gastrointestinal 4/8
	Fall 3/6	fever 0/2
	Unspecified cardiorespiratory 0/5	Fall 0/2
	fever 0/3	
	hypertension 2/2	
	weakness 0/1	
	hyperglycemia 0/1	
	hyponatremia 0/1	
III	**Grade 3 adverse events 19/162**	**Grade 3 adverse events 1/139**
	hospitalization 0/50	hospitalization 0/53
	unspecified health/medical problems leading to withdrawal 2/31	unspecified health/medical problems leading to withdrawal 0/38
	dizziness 5/20	Deep vein thrombosis 0/14
	cancer 0/16	cancer 0/14
	Deep vein thrombosis 5/15	dizziness 0/6
	infection 0/5	infection, 0/3
	Non-cardiac-related chest pain 3/4	bone pain 0/3
	pulmonary embolus 0/3	pulmonary embolus 0/2
	Pneumonia 0/3	Non-cardiac-related chest pain 1/2
	spinal stenosis 1/2	percutaneous coronary intervention 0/1
	stroke 0/2	fall related fracture 0/1
	Hyperthyoridim 1/2	acute myocardial infraction 0/2
	fall related fracture 0/2	
	COPD 0/2	
	pathological fracture 0/1	
	anemia 0/1	
	dehydration 1/1	
	Syncope 1/1	
	neutropenia 0/1	
IV	**Grade 4 adverse events 0/0**	**Grade 4 adverse events 0/0**
	Nil	Nil
V	**Grade 5 adverse events 0/37**	**Grade 5 adverse events 0/34**
	death 0/37	death 0/34

Adverse events were categorized based on the Common Terminology Criteria AEs as follows:

Grade 1: Characterized by asymptomatic or minor symptoms

Grade 2: Involving moderate symptoms, requiring minimal, local, or non-invasive intervention, and limiting age-appropriate instrumental activities of daily living

Grade 3: Marked by severe or medically significant effects that are not immediately life-threatening

Grade 4: Associated with life-threatening consequences, requiring urgent intervention

Grade 5: Death

Total adverse events description includes all adverse events (both exercise- and non-exercise related)

Adverse events lacking severity information were categorized as Grade 3 or higher if they resulted in study withdrawal

Instances labeled as "cancer" did not include specific details regarding whether the withdrawals were attributed to cancer progression, recurrence, or the emergence of other cancer types

### Adverse events in usual care participants

Among the 2125 participants allocated to usual care, 86 exercise-related adverse events out of 426 total adverse events were reported. Grade 1 adverse events were the most frequent, with 79 events, including musculoskeletal pain/myalgia (n = 33) and musculoskeletal discomfort (n = 22). Only 6 grade 2 events were reported, primarily unspecified minor symptoms (n = 2) and gastrointestinal issues (n = 4). The majority of severe adverse events were grade 3, totaling 139, with the most common being hospitalizations and unspecified health/medical problems leading to withdrawal (n = 38). Similar to the exercise group, no grade 4 events were reported, but there were 34 grade 5 events, all of which were deaths, and unrelated to the intervention.

### Health outcomes: assessment of outcomes

An overview of the instruments and methods used to evaluate various health outcomes across the 87 analyzed studies is presented in Table 4. Aerobic capacity was assessed in 49 studies, with the 6-min walk test being the most frequently used method (47%), followed by VO2max or VO2peak assessments using a cycle ergometer (16%) and the modified Bruce treadmill protocol (14%). Health-related Quality of Life (HRQOL) was evaluated in 38 studies, with the EORTC QLQ-C30 being the predominant tool (55%), followed by the Functional Assessment of Cancer Therapy (31%) and the Medical Outcomes Study 36-item short-form survey (8%). Fatigue was assessed in 39 studies, most commonly using the EORTC-F (28%) and the Functional Assessment of Cancer Therapy-Fatigue scale (21%). Strength was measured in 34 studies, with the handgrip dynamometer being the primary tool (58%). Anxiety was assessed in 14 studies, with the Hospital Anxiety and Depression Scale being the most commonly used tool (65%), and depression was evaluated in 17 studies, where the same scale was used in 53% of cases. The variety of instruments employed reflects the diverse approaches to evaluating these critical health outcomes in clinical research.

**Table 4:** Overview of results and methods in analysed studies (n = 87)

Outcome	Instrument/methods and number of studies
Cardiorespiratory fitness (n = 49)	6-min walk test, n = 23, (47%) VO2max or VO2peak assessed using a cycle ergometer, n = 8, (16%) VO2peak testing using a modified Bruce treadmill protocol, n = 7, (14%) 400-m walk test, n = 5, (10%) 12-min walk test, n = 2, (4%) Sit-to-stand test, n = 2, (4%) 2-min walk test, n = 1, (2%) 2-min stair climb test, n = 1, (2%)
Health-related Quality of Life (n = 38)	EORTC QLRC30, n = 21, (55%) Functional Assessment of Cancer Therapy, n = 12, (31%) Medical Outcomes Study 36-item short-form survey, n = 3, (8%) EQ-5D, n = 1, (3%) Quality of Life Measure for Limb Lymphoedema, n = 1, (3%)
Moderate-Vigour Physical activity (n = 18)	IPAQ, n = 4, (22%) Community Health Model Activities Program for Seniors, n = 3, (17%) activPAL wearable monitor, n = 3, (17%) Moderate-Vigour Physical activity assessed with the ActiGraph®, n = 2, (12%) Accelerometers, n = 2, (12%) 7-Day Physical Activity Recall, n = 1, (5%) Godin Leisure-Time Exercise, n = 1, (5%) Active Australia Survey, n = 1, (5%) Self-reported (scale 1—4), n = 1, (5%)
Daily steps (n = 16)	Accelerometers, n = 6, (38%) Wearable activity monitor, n = 5, (31%) Pedometers, n = 3 (19%) Self-reported, n = 2, (12%)
Fatigue (n = 39)	EORTC-F, n = 11, (28%) Functional Assessment of Cancer Therapy-Fatigue, n = 8, (21%) Functional Assessment of Chronic Illness Therapy – Fatigue Scale, n = 4, (10%) Multidimensional Fatigue Inventory, n = 4, (10%) Brief Fatigue Inventory, n = 3, (8%) Piper Fatigue Scale, n = 2, (5%) Fatigue Symptom Inventory, n = 1, (3%) Cancer Fatigue Scale, n = 1, (3%) Fatigue Severity Scale, n = 1, (3%) NCCN Scale, n = 1, (3%) Pacient Health Questionaire, n = 1, (3%) Medical Outcomes Study 36-item short-form survey, n = 1, (3%) Visual Analogue Scale (0 – 10), n = 1, (3%)
Strength (n = 34)	Handgrip dynamometer, n = 20, (58%) Chest press 1-RM, n = 6, (18%) Leg press 1-RM, n = 2, (6%) Back-leg dynamometer, n = 2, (6%) Isometric bench with a strain gauge (Globus System), n = 2, (6%) Push-up test, n = 1, (3%) Number of chair stands achieved during 30 s, n = 1, (3%)
Anxiety (n = 14)	Hospital Anxiety and Depression Scale, n = 9, (65%) Beck Anxiety Inventory, n = 1,(7%) Generalised Anxiety Disorder Assessment, n = 1,(7%) General Health Questionnaire-12, n = 1,(7%) State Trait Anxiety Inventory, n = 1,(7%) Patient-Reported Outcomes Measurement Information System®, n = 1,(7%)
Depression (n = 17)	Hospital Anxiety and Depression Scale, n = 9, (53%) Beck Anxiety Inventory, n = 3,(17%) Generalised Anxiety Disorder Assessment, n = 1, (6%) General Health Questionnaire-12, n = 1, (6%) Patient-Reported Outcomes Measurement Information System®, n = 1, (6%) Center for Epidemiologic Studies Depression Scale, n = 1, (6%) The Zung Self-Rating Depression Scale, n = 1, (6%)
Physical function (n = 34)	EORTC-F, n = 16, (46%) Medical Outcomes Study 36-item short-form survey, n = 8, (24%) Functional Assessment of Cancer Therapy, n = 7, (21%) Disabilities of the Arm, Shoulder and Hand Questionaire, n = 1, (3%) Unspecified three tests, n = 1, (3%) Patient-Reported Outcomes Measurement Information System®, n = 1, (3%)
Dyspnea (n = 17)	EORTC-D, n = 15, (88%) San Diego Shortness of Breath Questionnaire, n = 2, (12%)
Pain (n = 27)	EORTC-P, n = 14, (51%) Medical Outcomes Study 36-item short-form survey, n = 6, (22%) Visual Analogue Scale (0 – 10), n = 3, (11%) Chemotherapy-Induced Peripheral Neuropathy Questionaire, n = 1, (4%) Visual Analogue Scale (0 – 100), n = 1, (4%) Brief Pain Inventory-Short Form, n = 1, (4%) Worst Pain Intensity Scale, n = 1, (4%)
Sleep/Insomnia (n = 17)	EORTC-I, n = 11, (64%) Pittsburgh Sleep Quality Index, n = 4, (24%) Patient-Reported Outcomes Measurement Information System®, n = 1, (6%) General Sleep Disturbance Scale, n = 1, (6%)

1-RM, one repetition maximum; IPAQ, International Physical Activity Questionnaire; NCCN Scale, National Comprehensive Cancer Network Distress Thermometer; EORTC QLQ-C30, European Organisation for Research and Treatment of Cancer Core Quality of Life Questionnaire; EORTC, European Organisation for Research and Treatment of Cancer; EORTC-F, EORTC Fatigue Scale; EORTC-D, EORTC Dyspnea Scale; EORTC-P, EORTC Pain Scale; EORTC-I, EORTC Insomnia Scale; EQ-5D, EuroQol 5-Dimension Health Questionnaire; VO2peak, Peak oxygen uptake.

### Adverse events report quality

The quality of adverse event reporting was evaluated across 48 trials [11, 12, 26, 29, 30, 34–36, 38, 40, 42, 44–46, 48–50, 52, 56–58, 62, 63, 65, 69, 71, 73, 74, 81, 83, 87, 90, 93–96, 102, 105, 108, 120–122, 124, 130, 131, 133, 134, 143]. The median reporting score was 6.0 criteria (IQR: 3.0). The highest score achieved was 9 criteria (n = 3), while the lowest was 0 criteria (n = 1). Criterium 8b was the most frequently reported, appearing in 38 trials (79%). In contrast, Criterium 7 and Criterium 8d were reported in only two trials (4%) (Table 5 and Supplementary File 5).

**Table 5:** Evaluation of adverse event reporting criteria (n = 48)

Criteria	Reported, n (%)
1. In the title or abstract, trial states that safety or adverse events were assessed?	20 (41.6)
2. In the introduction, trial states that safety or adverse events were assessed?	9 (18.8)
Methods	
3a. Trial lists and defines all adverse events that were assessed	6 (12.5)
3b. Trial specifies instruments that were used to assess adverse events	9 (18.8)
4a. Trial describes how adverse events were collected	29 (60.4)
4b. Trial describes when adverse events were collected	25 (50.1)
4c. Trial describes attribution method	32 (66.7)
5. Trial describes how adverse events were analysed	13 (27.1)
Results	
6a. Trial reports number of participants discontinuing/withdrawing due to adverse events per arm	12 (25.0)
6b. Trial describes adverse events leading to patient discontinuation or withdrawal	11 (22.9)
7. Trial provides denominators used for each analysis of adverse events	2 (4.2)
8a. Trial reports adverse events per severity per arm	21 (43.8)
8b. Trial reports adverse events type per arm	38 (79.2)
8c. Trial reports adverse events seriousness per arm	14 (29.2)
8d. Trial reports how recurrent events were handled	2 (4.2)
Discussion	
10. Trial provide a balanced discussion of benefits and harms	16 (33.3)

To obtain more detailed data on exercise-related adverse events, we contacted corresponding authors from selected studies where reporting was incomplete or unclear. Of the authors contacted, two provided additional detailed information about exercise-related adverse events. Specifically, out of nine contacted authors, two responded, clarifying the nature and severity of exercise-related adverse events in their respective studies.

## Discussion

The findings from our systematic review indicate that home-based exercise training is generally safe, feasible, and beneficial for people diagnosed with cancer. Feasibility was demonstrated by the majority of studies meeting the pre-defined thresholds for recruitment, withdrawal, and adherence rates, highlighting the practicality of these interventions. However, variability across studies underscores the need to address barriers to implementation and optimize feasibility outcomes. Adverse events related to exercise or physical activity were relatively rare, occurring in less than 3.2% of participants, and were typically minor, reflecting normal physiological responses to exercise rather than serious health concerns. This aligns with prior research [3, 20, 21, 148], which has also shown a low incidence of adverse events in cancer populations engaging in exercise, though such studies often underrepresented individuals with advanced stages of cancer.

This review included both RCTs and single-arm trials to provide a comprehensive evaluation of feasibility, safety, and health-related outcomes of home-based exercise interventions. By including single-arm trials, we were able to capture valuable feasibility metrics, such as recruitment, retention, adherence, and acceptability, which are critical for understanding the practicality and success of these interventions. Future research should continue to leverage both randomized and single-arm designs to explore feasibility outcomes further and refine intervention strategies.

This review assessed feasibility based on pre-defined thresholds: recruitment rate > 25%, withdrawal rate < 25%, and adherence rate > 75%, as established in prior literature [21–23]. While most studies met these criteria, variability in adherence was a notable limitation, with 36.2% of studies failing to meet the adherence threshold. These findings underscore the importance of addressing participant barriers and enhancing adherence strategies in future interventions, such as incorporating telecoaching or personalized support systems.

Recruitment rates in the included studies varied widely, reflecting the diverse challenges associated with engaging cancer survivors in home-based exercise interventions. For instance, Ergun et al. (2013) reported a high recruitment rate of 93.8%, attributable to their inclusion of participants who had completed treatment and targeted a highly motivated population undergoing structured interventions [49]. Conversely, Bland et al. (2019) observed a lower recruitment rate of 47%, citing logistical barriers and limited access to exercise resources as significant challenges [33]. These findings highlight that recruitment success often depends on factors such as cancer type, stage, and participant demographics, underscoring the need for tailored outreach strategies.

In terms of participant retention, the overall withdrawal rate across studies averaged 13.7%. Higher withdrawal rates were often linked to lower-quality studies, as seen in Stuecher et al. (2019), which reported a 27.3% withdrawal rate, likely due to insufficient participant engagement and support during the intervention [123]. Conversely, studies with robust designs and participant support systems, such as Edbrooke et al. (2022), achieved zero withdrawals, demonstrating the critical role of ongoing supervision and tailored communication [48]. Health-related reasons, including treatment side effects and cancer progression, were the most frequently reported causes of withdrawal, emphasizing the importance of flexible and adaptive intervention designs to accommodate participants' evolving health needs.

Adherence to prescribed exercise regimens also exhibited significant variability, with an average adherence rate of 76.2%. Yee et al. (2019) exemplified optimal adherence with a 100% adherence rate, achieved through the use of comprehensive tracking tools and real-time feedback mechanisms [137]. However, adherence challenges were prevalent in studies with limited participant support, with rates as low as 50% in some cases. Adherence emerged as the most common unmet feasibility criterion, affecting 36% of studies in this review. This underscores the critical need for strategies such as telecoaching, individualized program adjustments, and digital health tools to enhance engagement and mitigate logistical barriers.

However, it is important to note that approximately half of the studies we reviewed did not report on the detailed occurrence or absence of adverse events methodology, highlighting a significant gap in the documentation and monitoring of exercise-related risks. In studies that did report adverse events, the details were often insufficient, lacking comprehensive descriptions of how these events were monitored and recorded. This observation is consistent with previous findings, which revealed that less than half of the reviewed studies documented adverse events, with the majority of those studies reporting no significant harm from exercise [149].

It is important to interpret the findings on safety with caution. Although adverse events were reported in less than 3.2% of participants, this low rate likely reflects underreporting rather than definitive evidence of safety. As noted in previous literature, adverse event reporting in the exercise oncology field is inconsistent and often incomplete [149]. This highlights the urgent need for standardized frameworks, such as the CONSORT extensions for harms, to improve the documentation and reporting of safety outcomes. Future studies should prioritize detailed monitoring and reporting of adverse events to provide a more accurate assessment of safety.

The variability in study designs, participant populations, and outcome measures also limited the potential for quantitative synthesis. While a meta-analysis was considered, the substantial heterogeneity in study designs, participant populations, outcome measures, and reporting practices precluded meaningful quantitative synthesis. Instead, we focused on providing a comprehensive qualitative synthesis to identify overarching trends and key challenges in the safety and feasibility of home-based exercise interventions. Our review also highlighted that 41.9% of studies did not meet at least one feasibility criterion, with adherence being the most common limitation (65.4% of these studies). This finding underscores the critical need to develop targeted strategies to enhance adherence, such as telecoaching or individualized support, which were underrepresented in the reviewed studies.

The results emphasize the necessity for standardized protocols in recording and reporting adverse events in exercise-related cancer trials. Although severe adverse events were minimal within the exercise intervention groups, this underscores the importance of conducting thorough health and medical assessments before prescribing exercise programs. Additionally, personalized exercise plans and patient education are crucial to ensure that participants can recognize and appropriately respond to any adverse events that may arise during their exercise regimen [150]. To address feasibility challenges, future research should include pilot studies prior to larger RCTs. These pilots could test intervention strategies, particularly adherence-focused approaches, to ensure the feasibility of achieving desired outcomes, such as improved aerobic capacity—a common primary outcome in exercise interventions.

These measures will not only enhance the safety of exercise interventions but also contribute to the broader understanding of exercise's role in cancer care, particularly in home-based settings where direct supervision is limited.

### Recruitment challenges and strategies

Recruitment to exercise oncology trials remains a significant challenge, as evidenced by the wide variability in recruitment rates observed in our review. This is consistent with the findings of Reynolds et al. (2023), who reported a median recruitment rate of 38% across various trials, with rates ranging from as low as 0.52% to 100% [151]. Such variability underscores the difficulty in engaging cancer survivors in exercise interventions, despite the well-documented benefits.

The type of cancer and the recruitment strategies employed play a crucial role in these rates [152, 153]. For instance, trials recruiting prostate cancer patients reported higher recruitment rates compared to those targeting colorectal cancer patients [151]. Active recruitment strategies, such as direct outreach by healthcare professionals, were associated with higher recruitment rates, suggesting that personalized approaches may be more effective in overcoming initial participation barriers [153, 154].

However, patient-oriented barriers, such as lack of interest, logistical challenges like distance and transportation, and difficulties in contacting potential participants, remain significant obstacles [155–157]. These barriers highlight the need for more tailored recruitment strategies that address the specific concerns and circumstances of cancer survivors. Enhanced public and patient involvement (PPI) in the design of recruitment strategies could help to align these efforts more closely with patient needs and preferences, thereby improving overall recruitment success [158–160].

### Adherence and completion rates

Adherence to exercise protocols in home-based interventions is critical for achieving the desired health outcomes. The studies reviewed reported varying levels of adherence, with some showing high adherence rates of 71–88% [161]. However, adherence can be influenced by several factors, including the design of the exercise program, participant motivation, and the clarity of adherence definitions used in the studies.

Adherence was the most frequently unmet feasibility criterion, with nearly 36% of studies failing to reach the threshold (> 75%). This highlights a significant barrier to intervention success and underscores the importance of individualized approaches, such as telecoaching, real-time feedback, and flexible exercise designs. The integration of these strategies into future trials may improve adherence, especially in populations that face logistical or motivational challenges.

Adherence was generally measured as the percentage of completed prescribed exercises or the number of sessions per week, often monitored through training diaries or digital platforms. High adherence was associated with significant improvements in physical outcomes, such as aerobic fitness and strength, particularly in studies involving prostate cancer patients undergoing androgen deprivation therapy, where adherence rates were around 80% (Toohey et al., 2022) [162]. However, the impact of adherence on other outcomes, such as quality of life and fatigue, was less consistent, suggesting that while adherence is crucial, it is not the only factor influencing the effectiveness of exercise interventions [163].

Completion rates were similarly varied, with some studies reporting lower adherence due to clinical issues, personal challenges, and motivational factors. For example, Falz et al. (2022) found that reductions in adherence were often linked to treatment side effects and personal motivations [164]. These findings underscore the need for exercise programs that are flexible and responsive to the changing health status and personal circumstances of cancer survivors.

### The role of mobile health and digital health technologies

The integration of mobile health and digital health technologies into home-based exercise interventions has shown considerable promise in enhancing both adherence and completion rates [165–168]. Digital tools offer several advantages, particularly in a home-based setting where participants lack direct supervision [169, 170]. They enable real-time tracking of exercise performance, provide personalized feedback, and offer motivational support through reminders and progress updates [171, 172].

For instance, the pilot study by Moraitis et al. (2022) on colorectal cancer survivors demonstrated that a mobile health-supported home-based exercise intervention could achieve high adherence and completion rates, with a reported 88.6% workout completion rate and 100% retention over a 12-week period [173]. Participants provided positive feedback on the ease of use, increased motivation, and autonomy offered by the mobile health platform. However, adherence to specific exercise intensities, such as those required in high-intensity interval training, was lower, indicating that while digital tools can support adherence, the design of the exercise program itself must be carefully considered to match participant capabilities and preferences.

The use of mobile health technologies also facilitates comprehensive data collection, allowing for more accurate monitoring and adjustments to exercise regimens in real-time [174]. This capability is crucial for personalizing interventions and ensuring they remain safe and effective for cancer survivors, particularly those undergoing intensive treatments like chemotherapy [175]. As shown in the studies reviewed, digital platforms can enhance engagement, but their effectiveness depends on how well they are integrated into the broader intervention strategy and how they address the specific needs of the participant population.

### The need for standardized reporting and evaluation

Despite the promising potential of home-based and mobile health-supported exercise interventions, the variability in recruitment, adherence, and completion rates highlights the need for more standardized approaches to reporting and evaluating these metrics. Inconsistent definitions and tracking methods make it difficult to compare outcomes across studies and to identify best practices.

Thomsen et al. (2022) emphasized the importance of standardized adverse event reporting, particularly in exercise trials involving cancer patients undergoing systemic treatment [20]. Their findings indicated a higher risk of certain serious adverse events in exercise groups, although the overall evidence was of very low certainty. This underscores the need for rigorous monitoring and clear reporting protocols, such as the Exercise Harms Reporting Method (ExHaRM) proposed by Spence et al. (2022) [176]. Adopting such standardized protocols would improve the quality and comparability of data across studies, providing a clearer understanding of the risks and benefits of exercise interventions in cancer populations.

### Future directions

To enhance the effectiveness of home-based exercise interventions, future research should focus on refining recruitment strategies to reach a broader and more diverse population, particularly those typically underrepresented in clinical trials. This involves identifying and addressing barriers such as logistical challenges, health disparities, and a lack of awareness regarding the benefits of exercise. Efforts should include tailored approaches, pre-exercise assessments, educational resources, and low-intensity starter programs designed to build confidence and minimize risks.

Improving adherence through personalized and flexible program designs and exploring the integration of mobile health tools tailored to cancer survivors' needs are also critical areas of development. Digital health technologies have the potential to provide guidance, monitor progress, and enhance motivation, especially for individuals lacking direct supervision during home-based interventions. However, the feasibility and effectiveness of these technologies across diverse cancer populations warrant further investigation.

Additionally, pragmatic studies employing representative samples are needed to evaluate 'real-world' home-based exercise interventions, especially among underrepresented subgroups, such as specific cohorts of breast cancer survivors [177]. Exploring motivations behind participation in exercise trials—and regular physical activity outside structured programs—could provide valuable insights into strategies for maintaining physical activity among cancer survivors both during and after treatment.

Moreover, future studies should explicitly evaluate and report intervention outcomes according to cancer stage and treatment status. Clearly differentiating these variables will enable more precise interpretations of safety and feasibility metrics, and help tailor interventions to meet specific patient needs.

Finally, the establishment of standardized definitions and consistent methods for assessing adherence, completion, and adverse events will be essential to advancing the field [178]. Addressing these methodological challenges and actively involving previously underrepresented populations in study designs and clinical guidelines will help optimize home-based exercise interventions, ensuring their safety, accessibility, and effective integration into standard cancer care.

### Limitations

While this systematic review provides valuable insights into the feasibility, safety, and effectiveness of home-based exercise interventions for cancer survivors, several limitations must be acknowledged. Firstly, the heterogeneity of the included studies, particularly in terms of cancer types, stages, and exercise protocols, limits the generalizability of the findings. The variability in adherence and completion rates across studies, along with inconsistent definitions and reporting methods, further complicates direct comparisons and synthesis of results. Additionally, the reliance on self-reported adherence and the lack of standardized adverse event reporting protocols may have led to underreporting or misclassification of exercise-related risks. The integration of technologies, while promising, also varied widely in its application and effectiveness, which might affect the consistency of outcomes. Importantly, the current evidence base may not adequately represent certain populations, such as those with advanced-stage cancer, significant comorbidities, or those from underserved communities, which limits the applicability of these findings to such groups. As a result, we cannot provide specific recommendations for these populations and emphasize the need for future research to specifically target these underrepresented groups to better understand their needs and potential risks associated with home-based exercise interventions. Future research should aim to standardize reporting methods and include more diverse and representative cancer populations to enhance the applicability of findings across different survivor groups. An additional limitation is that we included studies irrespective of participants' treatment status, leading to heterogeneity in outcomes and potentially masking important differences in feasibility, adherence, and safety among participants undergoing active treatment versus those who completed treatment.

While this systematic review provides valuable insights into the feasibility, safety, and effectiveness of home-based exercise interventions for cancer survivors, several limitations must be acknowledged. First, the heterogeneity among included studies regarding cancer types, stages, and exercise protocols limits the generalizability of the findings. Variability in adherence and completion rates across studies, combined with inconsistent definitions and reporting methods, further complicates direct comparisons and synthesis of results. Additionally, reliance on self-reported adherence and the absence of standardized protocols for adverse event reporting may have resulted in underreporting or misclassification of exercise-related risks. Although digital technologies are promising, their varied application and effectiveness across studies may also contribute to inconsistent outcomes.

Importantly, the current evidence base may inadequately represent populations such as those with advanced-stage cancer, significant comorbidities, or individuals from underserved communities, limiting the applicability of these findings. Consequently, specific recommendations for these populations are difficult to provide, emphasizing the necessity of future research explicitly targeting these groups.

Furthermore, our review included studies irrespective of participants' treatment status (active treatment versus post-treatment), potentially introducing variability and masking important differences in feasibility, adherence, and safety outcomes. Future research should clearly stratify and report outcomes by participants' treatment status to better inform clinical recommendations and intervention design. Standardizing reporting practices and including more diverse and representative cancer populations will significantly enhance the applicability and clinical relevance of future findings.

## Conclusion

This systematic review highlights the significant potential of home-based exercise interventions, as an integral part of cancer care, particularly for survivors. The findings suggest that these interventions are generally safe, feasible, and beneficial for cancer survivors. However, the variability in recruitment, adherence, and completion rates emphasizes the importance of addressing practical barriers to participation and implementation. While cancer-specific exercise guidelines already advocate for tailored and patient-centered approaches, further research is needed to translate these recommendations into effective, real-world strategies that enhance accessibility and adherence across diverse cancer populations. Additionally, the development of standardized protocols for monitoring and reporting outcomes remains a priority. By addressing these challenges, the integration of home-based exercise into routine cancer care can be optimized, ultimately improving the long-term health and well-being of cancer survivors.

## Supplementary Information

Below is the link to the electronic supplementary material.

Supplementary file1 (DOCX 15 KB)

Supplementary file2 (DOCX 17 KB)

Supplementary file3 (XLSX 22 KB)

Supplementary file4 (DOCX 34 KB)

Supplementary file5 (XLSX 22 KB)

Supplementary file6 (XLSX 22 KB)

Supplementary file7 (PNG 7850 KB)

Supplementary file8 (PNG 141 KB)

## Data Availability

No datasets were generated or analysed during the current study.
