# Supplementary material for: Feasibility, safety, and adherence of home-based exercise interventions in people diagnosed with cancer: a systematic review
Source: J Cancer Surviv. 2025 Mar 22;20(Suppl 1):982–1003. doi: 10.1007/s11764-025-01778-5 (PMC13676528; doi:10.1007/s11764-025-01778-5)
Supplement: Supplementary file 1 [file 11764_2025_1778_MOESM1_ESM.docx]

Table 1. Search Strategies for the Used Electronic Databases

| Electronic databases | Search term |
| --- | --- |
| Pubmed and Medline | “Cancer”[Mesh] OR cancer [tiab] AND “Exercise”[Mesh] OR Exercise*[tiab] OR physical activit*[tiab] OR resistence training [tiab] OR aerobic [tiab] OR sport*[tiab] OR walk*[tiab] OR move*[tiab] OR active [tiab] OR lifestyle*[tiab] OR performance [tiab] OR training intervention*[tiab] OR sedentary behaviour [tiab]  Filters: English |
| CINAHL, ProQuest Health and Medical Complete, Science Direct and SPORTDiscus | (MH “Cancer”) OR (MH “Exercise+”) OR TX(“exercise”) OR (MH “Therapeutic Exercise+”) OR (MH “Aerobic Exercises+”) OR (MH “Weight Training”) OR (MH “Resistance Training”) OR (MH “Exercise Intensity”) OR (MH “Sports Nutritional Sciences”) OR (MH “Exercise Adherence”) OR (MH “Physical Activity”) OR (MH “Physical  Endurance+”) OR (MH “Physical Performance”) OR (MH “Physical Fitness+”) OR (MH “Lifestyle”) OR (MH “Sedentary”) |
