# Supplementary material for: Feasibility, safety, and adherence of home-based exercise interventions in people diagnosed with cancer: a systematic review
Source: J Cancer Surviv. 2025 Mar 22;20(Suppl 1):982–1003. doi: 10.1007/s11764-025-01778-5 (PMC13676528; doi:10.1007/s11764-025-01778-5)
Supplement: Supplementary file 2 [file 11764_2025_1778_MOESM2_ESM.docx]

# Supplementary Table 2 Quality of adverse events reporting scoring system

| **Study section** | **CONSORT Harms item** | | **Scoring item *** | | |  |
| --- | --- | --- | --- | --- | --- | --- |
| Title/abstract | 1. | If the study collected data on harms and benefits, the title or abstract should so state | | 1. | In the title or abstract, the trial states that safety or adverse events were assessed. | |
| Introduction | 2. | If the study addresses both harms and benefits, the introduction should so state | | 2. | In the introduction, the trial states that safety or adverse events were assessed. | |
| Methods | 3. | List addressed adverse events with definitions for each (with attention, when relevant, to grading, expected vs. unexpected events, reference to standardized and validated definitions, and description of new definitions). | | 3a | Trial lists and defines all adverse events that were assessed. | |
|  |  |  |  | 3b. | Trial specifies instruments that were used to assess adverse events. | |
|  | 4. | Clarify how harms-related information was collected (mode of data collection, timing, attribution methods, intensity of ascertainment, and harms-related monitoring and stopping rules, if pertinent). | | 4a. | Trial describes how adverse events were collected. | |
|  |  |  |  | 4b. | Trial describes when adverse events were collected. | |
|  |  |  |  | 4c. | Trial describes attribution method. | |
|  | 5. | Describe plans for presenting and analyzing information on harms (including coding, handling of recurrent events, specification of timing issues, handling of continuous measures, and any statistical analyses). | | 5. | Trial describes how adverse events were analysed. | |
|  |  |  |  |  |  | |
| Results | 6. | Describe for each arm the participant withdrawals that are due to harms and their experiences with the allocated treatment. | | 6a. | Trial reports number of participants discontinuing/withdrawing due to adverse events per arm. | |
|  |  |  |  | 6b. | Trial describes adverse events leading to patient discontinuation or withdrawal. | |
|  | 7. | Provide the denominators for analyses on harms. | | 7. | Trial provides denominators used for each analysis of adverse events. | |
|  | 8. | Present the absolute risk per arm and per adverse event type, grade, and seriousness, and present appropriate metrics for recurrent events, continuous variables, and scale variables, whenever pertinent. | | 8a. | Trial reports adverse events per severity | |
|  |  |  |  | 8b. | Trial reports adverse events type. | |
|  |  |  |  | 8c. | Trial reports adverse events seriousness | |
|  |  |  | | 8d | Trial reports how recurrent events were handled. | |
|  |  |  | |  |  | |
|  | 9. | Describe any subgroup analyses and exploratory analyses for harms. | | 9. | - | |
| Discussion | 10. | Provide a balanced discussion of benefits and harms with emphasis on study limitations, generalizability, and other sources of information on harms. | | 10. | The study provides a balanced discussion of benefits and harms in the discussion. | |

*An item was given a score of 1 if it was reported and a score of 0 if it was unclearly reported or not reported. The quality of adverse events score was the summed score of all items, with each item being equally weighted.
