# Supplementary material for: Feasibility, safety, and adherence of home-based exercise interventions in people diagnosed with cancer: a systematic review
Source: J Cancer Surviv. 2025 Mar 22;20(Suppl 1):982–1003. doi: 10.1007/s11764-025-01778-5 (PMC13676528; doi:10.1007/s11764-025-01778-5)
Supplement: Supplementary file 3 [file 11764_2025_1778_MOESM4_ESM.docx]

Supplementary content: Ratings of all studies included in systematic review using the PEDro scale (n=120)

| First author, year of publication | PEDro Scale item number | | | | | | | | | | | Total score | Quality |  |
| --- | --- | --- | --- | --- | --- | --- | --- | --- | --- | --- | --- | --- | --- | --- |
|  | 1 | 2 | 3 | 4 | 5 | 6 | 7 | 8 | 9 | 10 | 11 |  |  |  |
| Abo, 2018 [24] | Y | Y | Y | Y | N | N | N | Y | N | Y | Y | 6 | High |  |
| Alibhai,2014 [25] | Y | Y | Y | Y | N | N | N | Y | Y | Y | Y | 7 | High |  |
| Alibhai,2019 [26] | Y | Y | Y | Y | N | N | Y | N | N | Y | Y | 6 | High |  |
| Allen, 2022 [27] | Y | Y | Y | Y | N | N | Y | Y | Y | Y | Y | 8 | High |  |
| Ammitzbøll,2019 [10] | Y | Y | N | Y | N | N | Y | N | Y | Y | Y | 6 | High |  |
| Backman,2014 [28] | Y | Y | N | Y | N | N | N | Y | N | Y | Y | 5 | Low |  |
| Bade,2021 [29] | Y | Y | Y | Y | N | N | N | Y | N | Y | Y | 6 | High |  |
| Bao,2020 [30] | Y | Y | N | Y | N | N | N | Y | Y | Y | Y | 6 | High |  |
| Baruth,2015 [31] | Y | Y | N | Y | N | N | N | Y | N | N | Y | 4 | Low |  |
| Beidas,2014 [32] | Y | Y | Y | Y | N | N | N | Y | N | Y | Y | 6 | High |  |
| Bland, 2019 [33] | Y | Y | Y | Y | N | N | N | Y | N | Y | Y | 6 | High |  |
| Blarigan,2019 [34] | Y | Y | N | Y | N | N | N | Y | N | Y | Y | 5 | Low |  |
| Brown, 2023 [35] | Y | Y | N | Y | N | N | N | Y | N | Y | Y | 5 | Low |  |
| Brown,2017 [36] | Y | Y | N | Y | N | N | Y | Y | Y | Y | Y | 7 | High |  |
| Buchan,2016 [37] | Y | Y | N | Y | N | N | N | Y | Y | Y | Y | 6 | High |  |
| Caan, 2024 [141] | Y | Y | Y | Y | N | N | N | Y | N | Y | Y | 6 | High |  |
| Coleman, 2008 [38] | Y | Y | N | N | N | N | N | Y | N | Y | Y | 4 | Low |  |
| Cornette, 2015 [39] | N | Y | N | Y | N | N | N | N | Y | Y | Y | 5 | Low |  |
| Dalla-Via 2021 [40] | Y | Y | N | Y | N | N | N | Y | Y | Y | Y | 6 | High |  |
| De Lazzani [142] | Y | Y | N | Y | N | N | Y | Y | N | Y | Y | 6 | High |  |
| Demark-Wahnefried, 2008 [41] | Y | Y | N | Y | N | N | N | Y | N | Y | Y | 5 | Low |  |
| Demark-Wahnefried, 2012 [42] | Y | Y | N | Y | N | N | N | Y | Y | N | Y | 5 | Low |  |
| Dhawan, 2019 [43] | Y | Y | Y | Y | N | N | N | Y | Y | Y | Y | 7 | High |  |
| Dhillon,2017 [44] | Y | Y | N | Y | N | N | N | N | Y | Y | Y | 5 | Low |  |
| Dodd, 2010 [45] | Y | Y | N | Y | N | N | N | Y | Y | Y | N | 5 | Low |  |
| Eakin, 2012 [46] | Y | Y | N | Y | N | N | N | Y | Y | Y | Y | 6 | High |  |
| Edbrooke, 2019 [47] | Y | Y | Y | Y | N | N | Y | N | Y | Y | Y | 7 | High |  |
| Edbrooke,2022 [48] | Y | Y | Y | N | N | N | Y | N | Y | N | Y | 5 | Low |  |
| Ergun, 2013 [49] | Y | Y | N | Y | N | N | Y | Y | N | Y | Y | 6 | High |  |
| Freedland,2019 [50] | Y | Y | N | Y | N | N | Y | Y | N | Y | Y | 6 | High |  |
| Galiano-Castillo,2017 [51] | Y | Y | Y | Y | N | N | Y | Y | Y | Y | Y | 8 | High |  |
| Galvao, 2014 [52] | Y | Y | N | Y | N | N | N | Y | Y | Y | Y | 6 | High |  |
| Gehring,2018 [53] | Y | Y | Y | Y | N | N | Y | Y | Y | Y | Y | 8 | High |  |
| Gorzelitz,2023 [54] | Y | Y | N | Y | N | N | N | Y | Y | N | Y | 5 | Low |  |
| Granger, 2012 [55] | Y | Y | Y | Y | N | N | Y | N | Y | Y | Y | 7 | High |  |
| Granger, 2024 [143] | Y | Y | Y | Y | N | N | Y | N | Y | Y | Y | 7 | High |  |
| Ha,2023 [56] | Y | Y | N | Y | N | N | Y | Y | N | Y | Y | 6 | High |  |
| Haines, 2010 [57] | Y | Y | Y | Y | N | N | N | N | N | Y | Y | 5 | Low |  |
| Harrison,2022 [58] | Y | Y | N | Y | N | N | Y | Y | N | Y | Y | 6 | High |  |
| Heimen, 2022 [59] | Y | Y | N | N | N | N | N | N | Y | Y | N | 3 | Low |  |
| Hoffman,2016 [60] | Y | Y | Y | Y | N | N | Y | Y | N | Y | Y | 7 | High |  |
| Huberty,2019 [61] | Y | Y | N | Y | N | N | N | N | N | Y | Y | 4 | Low |  |
| Husebo, 2014 [62] | Y | Y | Y | Y | N | N | N | Y | N | Y | Y | 6 | High |  |
| Chan,2020 [63] | Y | Y | N | Y | N | N | N | N | N | Y | Y | 4 | Low |  |
| Chen,2015 [64] | Y | Y | Y | Y | N | N | N | Y | Y | Y | Y | 7 | High |  |
| Cheville,2013 [65] | Y | Y | Y | Y | N | N | N | N | Y | Y | Y | 6 | High |  |
| Cheville,2019 [66] | Y | Y | Y | Y | N | N | Y | Y | Y | Y | Y | 8 | High |  |
| Chuang,2017 [67] | Y | Y | Y | Y | N | N | N | Y | N | Y | Y | 6 | High |  |
| Jarden, 2009 [68] | Y | Y | N | Y | N | N | N | N | Y | Y | Y | 5 | Low |  |
| Jarden, 2016 [69] | Y | Y | N | Y | N | N | N | Y | N | Y | Y | 5 | Low |  |
| Johansson, 2013 [70] | Y | Y | N | Y | N | N | Y | Y | Y | Y | Y | 7 | High |  |
| Johansson,2014 [71] | Y | Y | N | Y | N | N | N | N | N | Y | Y | 4 | Low |  |
| Kanzawa-Lee, 2022 [72] | Y | Y | N | Y | N | N | Y | Y | Y | Y | Y | 7 | High |  |
| Karlsson,2019 [73] | Y | Y | Y | Y | N | N | Y | Y | N | Y | Y | 7 | High |  |
| Karvinen, 2014 [11] | Y | Y | Y | Y | N | N | N | N | N | Y | Y | 5 | Low |  |
| Kenfield, 2019 [74] | Y | Y | N | Y | N | N | N | Y | Y | Y | Y | 6 | High |  |
| Kenfield, 2021 [75] | Y | Y | Y | N | N | N | N | N | N | N | Y | 3 | Low |  |
| Kilbreath,2020 [76] | Y | Y | Y | Y | N | N | Y | Y | Y | Y | Y | 8 | High |  |
| Kim,2016 [77] | Y | Y | Y | Y | N | N | Y | Y | Y | Y | Y | 8 | High |  |
| klecker, 2018 [78] | Y | Y | Y | Y | N | N | N | N | Y | Y | Y | 6 | High |  |
| Knoerl, 2022 [79] | Y | Y | N | Y | N | N | N | N | Y | Y | Y | 5 | Low |  |
| Lahart,2016 [80] | Y | Y | N | Y | N | N | N | Y | Y | Y | Y | 6 | High |  |
| Lam,2020 [81] | Y | Y | N | Y | N | N | N | Y | Y | Y | Y | 6 | High |  |
| Langlais,2023 [82] | Y | Y | Y | Y | N | N | N | Y | N | Y | Y | 6 | High |  |
| Larsen, 2019 [83] | Y | Y | N | Y | N | N | Y | N | Y | Y | Y | 6 | High |  |
| Lee,2018 [84] | Y | Y | N | Y | N | N | N | N | Y | Y | Y | 5 | Low |  |
| Ligibel, 2016 [85] | Y | Y | N | Y | N | N | Y | Y | N | Y | Y | 6 | High |  |
| Loudon,2014 [86] | N | Y | Y | Y | N | N | Y | N | N | Y | Y | 5 | Low |  |
| Lynch,2019 [87] | Y | Y | Y | Y | N | N | N | N | Y | Y | Y | 6 | High |  |
| Matthews, 2007 [88] | Y | Y | N | Y | N | N | N | N | Y | Y | Y | 5 | Low |  |
| Maurer, 2022 [89] | Y | Y |  | Y | N | N | N | Y | Y | Y | Y | 7 | High |  |
| Mikkelsen, 2022 [90] | Y | Y | N | Y | N | N | Y | Y | N | Y | Y | 6 | High |  |
| Minella, 2018 [91] | Y | Y | Y | Y | N | N | Y | N | N | Y | Y | 6 | High |  |
| Mock, 2005 [92] | Y | Y | Y | Y | N | N | N | Y | Y | N | Y | 6 | High |  |
| Moller,2020 [93] | Y | Y | N | Y | N | N | Y | N | N | Y | Y | 5 | Low |  |
| Morey, 2009 [94] | Y | Y | N | Y | N | N | Y | Y | N | Y | Y | 6 | High |  |
| Muller, 2021 [95] | Y | Y | N | Y | N | N | Y | Y | Y | Y | Y | 7 | High |  |
| Musanti, 2012 [96] | Y | Y | Y | Y | N | N | Y | N | N | Y | Y | 6 | High |  |
| Mutrie, 2007 [97] | Y | Y | Y | Y | N | N | Y | Y | Y | Y | Y | 8 | High |  |
| Naumann, 2012 [98] | Y | Y | N | Y | N | N | N | N | Y | Y | Y | 5 | Low |  |
| Ndjavera,2019 [99] | Y | Y | Y | Y | N | N | Y | N | Y | Y | Y | 7 | High |  |
| Nyrop,2017 [100] | Y | Y | N | Y | N | N | N | Y | Y | Y | Y | 6 | High |  |
| Occhi, 2021 [101] | N | Y | Y | Y | N | Y | Y | Y | Y | Y | Y | 8 | High |  |
| Oneil,2014 [102] | Y | Y | Y | Y | N | N | N | Y | Y | Y | Y | 7 | High |  |
| Onerup, 2024 [144] | Y | Y | N | N | N | N | Y | Y | N | Y | Y | Y | Low |  |
| Park, 2021 [103] | Y | Y | N | Y | N | N | N | Y | Y | Y | Y | 6 | High |  |
| Philips,2021 [104] | Y | Y | N | Y | N | N | N | Y | Y | Y | Y | 6 | High |  |
| Pinto,2021 [105] | Y | Y | Y | Y | N | N | Y | N | Y | Y | Y | 7 | High |  |
| Portella, 2008 [106] | Y | Y | N | Y | N | N | Y | N | N | Y | Y | 5 | Low |  |
| Porter,2019 [107] | Y | Y | Y | Y | N | N | N | N | Y | Y | Y | 6 | High |  |
| Qi [145] | Y | Y | N | Y | N | N | Y | N | N | Y | Y | 5 | Low |  |
| Ramos, 2023 [108] | Y | Y | N | Y | N | N | N | Y | Y | Y | Y | 6 | High |  |
| Rees Punia,2022 [109] | Y | Y | N | Y | N | N | N | N | N | Y | N | 3 | Low |  |
| Regers, 2009 [110] | Y | Y | Y | Y | N | N | N | Y | Y | Y | Y | 7 | High |  |
| Rief,2014 [111] | Y | Y | N | Y | N | N | N | N | N | Y | Y | 4 | Low |  |
| Sajit,2016 [112] | Y | Y | N | Y | N | N | Y | Y | N | Y | Y | 6 | High |  |
| Samuel, 2019 [113] | Y | Y | Y | Y | N | N | N | Y | N | Y | Y | 6 | High |  |
| Santa Mina, 2013 [114] | Y | Y | Y | Y | N | N | N | N | Y | Y | Y | 6 | High |  |
| Schmitz, 2009 [115] | Y | Y | Y | Y | N | N | Y | Y | N | Y | Y | 7 | High |  |
| Schmitz, 2019 [116] | Y | Y | N | Y | N | N | Y | N | Y | Y | Y | 6 | High |  |
| Schuler, 2017 [117] | Y | Y | N | Y | N | N | N | N | N | Y | Y | 4 | Low |  |
| Schumacher,2020 [118] | Y | Y | N | Y | N | N | Y | N | N | Y | Y | 5 | Low |  |
| Singh,2020 [119] | N | Y | N | Y | N | N | Y | N | N | Y | Y | 4 | Low |  |
| Solheim, 2017 [120] | Y | Y | N | Y | N | N | N | Y | N | Y | Y | 5 | Low |  |
| Stan,2016 [121] | Y | Y | N | Y | N | N | N | N | N | Y | Y | 4 | Low |  |
| Storck,2020 [122] | Y | Y | N | Y | N | N | N | Y | N | Y | Y | 5 | Low |  |
| Stuecher, 2019 [123] | Y | Y | N | N | N | N | N | N | N | Y | Y | 3 | Low |  |
| Sturgeon, 2022 [12] | Y | Y | N | Y | N | N | N | N | N | Y | Y | 4 | Low |  |
| Takemura, 2024 [146] | Y | Y | N | Y | N | N | N | Y | Y | Y | Y | 6 | High |  |
| Tsai, 2019 [124] | Y | N | N | Y | N | N | N | N | N | Y | Y | 3 | Low |  |
| Uhm,2017 [125] | N | N | Y | Y | N | N | N | Y | N | Y | Y | 4 | Low |  |
| Vallance,2015 [126] | Y | Y | Y | Y | N | N | N | N | Y | Y | Y | 6 | High |  |
| Valle, 2022 [127] | Y | Y | N | Y | N | N | N | Y | Y | Y | Y | 6 | High |  |
| Vallerand,2018 [128] | Y | Y | Y | Y | N | N | N | Y | Y | Y | Y | 7 | High |  |
| Van Blarigan [147] | Y | Y | N | Y | N | N | N | Y | Y | Y | Y | 6 | High |  |
| van Waart, 2015 [129] | Y | Y | N | Y | N | N | N | Y | Y | Y | Y | 6 | High |  |
| Villumsen,2019 [130] | Y | Y | Y | Y | N | N | Y | Y | Y | Y | Y | 8 | High |  |
| Vincent, 2020 [131] | Y | Y | N | Y | N | N | N | Y | Y | Y | Y | 6 | High |  |
| Wall,2017 [132] | Y | Y | Y | Y | N | N | N | N | Y | Y | Y | 6 | High |  |
| Wang, 2011 [133] | Y | Y | N | Y | N | N | N | Y | N | Y | Y | 5 | Low |  |
| Winters Stone,2021 [134] | Y | Y | N | Y | N | N | Y | Y | N | Y | Y | 6 | High |  |
| Wiskemann, 2019 [135] | Y | Y | N | Y | N | N | N | N |  | Y | Y | 5 | Low |  |
| Yang, 2010 [136] | Y | Y | N | Y | N | N | N | Y | N | Y | Y | 5 | Low |  |
| Yee,2019 [137] | Y | Y | Y | Y | N | N | N | Y | Y | Y | Y | 7 | High |  |
| Yeo, 2012 [138] | Y | Y | Y | Y | N | N | N | Y | Y | Y | Y | 7 | High |  |
| Zhang, 2018 [139] | Y | Y | Y | Y | N | N | Y | Y | Y | Y | Y | 8 | High |  |
| Zopf, 2021 [140] | Y | Y | N | N | N | N | Y | Y | N | Y | Y | 5 | Low |  |
| Pedro scale items: 1. Eligibility criteria; 2. Subjects randomly allocated; 4. Groups similar at baseline; 5. Subject blinding; 6. Therapist blinding; 7. Assessor blinding; 8. Outcome obtained from >85% of subjects; 9. Intention to treat; 10. Results of between-group comparisons; 11. Point and variability measures.  A score 6 or higher was considered high quality and trials receiving less than 6 were classified as low quality. | | | | | | | | | | | | | | |
